# Cumulative stress restricts niche filling potential of habitat‐forming kelps in a future climate

**DOI:** 10.1111/1365-2435.12977

**Published:** 2017-09-25

**Authors:** Nathan G. King, David C. Wilcockson, Richard Webster, Dan A. Smale, Laura S. Hoelters, Pippa J. Moore

**Affiliations:** ^1^ Institute of Biological, Environmental and Rural Sciences Aberystwyth University Aberystwyth UK; ^2^ Marine Biological Association of the United Kingdom The Laboratory Plymouth UK; ^3^ Centre for Marine Ecosystems Research School of Natural Sciences Edith Cowan University Joondalup WA Australia

**Keywords:** climate change, heat shock response, *Laminaria digitata*, *Laminaria ochroleuca*, leading edge, range shift, Trailing edge

## Abstract

1. Climate change is driving range contractions and local population extinctions across the globe. When this affects ecosystem engineers the vacant niches left behind are likely to alter the wider ecosystem unless a similar species can fulfil them.
2. Here, we explore the stress physiology of two coexisting kelps undergoing opposing range shifts in the Northeast Atlantic and discuss what differences in stress physiology may mean for future niche filling.
3. We used chlorophyll florescence (*F*_v_ */F*_m_) and differentiation of the heat shock response (HSR) to determine the capacity of the expanding kelp*, Laminaria ochroleuca*, to move into the higher shore position of the retreating kelp, *Laminaria digitata*. We applied both single and consecutive exposures to immersed and emersed high and low temperature treatments, replicating low tide exposures experienced in summer and winter.
4. No interspecific differences in HSR were observed which was surprising given the species’ different biogeographic distributions. However, chlorophyll florescence revealed clear differences between species with *L. ochroleuca* better equipped to tolerate high immersed temperatures but showed little capacity to tolerate frosts or high emersion temperatures.
5. Many patterns observed were only apparent after consecutive exposures. Such cumulative effects have largely been overlooked in tolerance experiments on intertidal organisms despite being more representative of the stress experienced in natural habitats. We therefore suggest future experiments incorporate consecutive stress into their design.
6. Climate change is predicted to result in fewer ground frosts and increased summer temperatures. Therefore, *L. ochroleuca* may be released from its summer cold limit in winter but still be prevented from moving up the shore due to desiccation in the summer. *Laminaria ochroleuca* will, however, likely be able to move into tidal pools. Therefore, only partial niche filling by *L. ochroleuca* will be possible in this system as climate change advances.

A plain language summary is available for this article.

## 1 INTRODUCTION

The world's oceans have warmed by 0.11°C per decade for the last 40 years while short term rapid increases in ocean and aerial temperatures (heatwaves) have also increased in frequency and duration (Christidis, Stott, Brown, Hegerl, & Caesar, 2005; Coumou & Rahmstorf, 2012; Hartmann, Tank, & Rusticucci, 2013; Lima & Wethey, 2012; Meehl & Tebaldi, 2004; Seneviratne et al., 2012a). Together, these two types of warming are resulting in the global redistribution of marine species (Burrows et al., 2011; Hobday et al., 2016; Poloczanska et al., 2013; Smale & Wernberg, 2013), altering the structure and functioning of entire ecosystems (Doney et al., 2012; Hawkins et al., 2009; Wernberg et al., 2016). In marine systems, organisms generally occupy the entirety of their “thermal niche” meaning marine populations are particularly responsive to warming (Sunday, Bates, & Dulvy, 2012). While local conditions can cause mosaic patterns of stress intensities in the intertidal (Helmuth et al., 2016; Lourenço et al., 2016), it is still range margins that are seen to be at the forefront of warming trends, resulting in poleward range expansions (leading edge) or contractions (trailing edge) (Chen, Hill, Ohlemüller, Roy, & Thomas, 2011; Hampe & Petit, 2005; Poloczanska et al., 2013). Understanding the physical determinants that set these boundaries is therefore critical for predicting the structure and functioning of ecosystems as climate change progresses (Gaston, 2003).

Kelps dominate shallow temperate rocky reefs where they support high levels of primary productivity and modify environmental conditions such as light (Wernberg, Kendrick, & Toohey, 2005), water flow (Rosman, Koseff, Monismith, & Grover, 2007), physical disturbance (Connell, 2003) and sedimentation rates (Eckman, Duggins, & Sewell, 1989), allowing rich assemblages to persist (Steneck et al., 2002). The biogeographic ranges of kelp species are largely controlled by temperature (Eggert, 2012; Lüning & tom Dieck, 1990). As such, increases in mean temperatures and heatwaves can alter macroalgal distributions (Edwards & Estes, 2006; Filbee‐Dexter, Feehan, & Scheibling, 2016; Smale & Wernberg, 2013; Smale, Wernberg, Yunnie, & Vance, 2015; Tuya et al., 2012; Voerman, Llera, & Rico, 2013), which can have catastrophic consequences for the associated ecosystems they underpin (Wernberg et al., 2013, 2016).

The British Isles is an important Northeast Atlantic biogeographical transition zone (Forbes, 1853). Along Britain's south coast, two structurally similar canopy‐forming kelps occur in sympatry at opposing edges of their distributional ranges. *Laminaria digitata,* a boreal species, approaches its trailing edge while *Laminaria ochroleuca,* a Lusitanian species, reaches its leading edge (Bartsch, Vogt, Pehlke, & Hanelt, 2013; Smale et al., 2015). Both coexist on moderately sheltered to moderately exposed shores where their vertical distributions overlap. *Laminaria digitata* dominates the low intertidal (<1 m above *Chart datum*) before being replaced by *L. ochroleuca* further down the shore (Hargrave, Foggo, Pessarrodona, & Smale, 2016). These species are currently undergoing opposing poleward range migrations, with *L. digitata* population declines observed along both sides of the English Channel, while *L. ochroleuca* has proliferated at its leading range edge (Raybaud et al., 2013; Smale et al., 2015). *Laminaria digitata* is a key ecosystem engineer (Schultze, Janke, Krüß, & Weidemann, 1990) and its loss could significantly impact wider community structure, unless another functionally similar species, such as *L. ochroleuca,* is able to move into the vacated habitat left behind.

The environmental conditions a species can survive in (fundamental niche) are often wider than where it is found (realised niche). The reason for this disparity is often due to interspecific competitive exclusion (Hutchinson, 1957, 1978). It is not known to what extent *L. ochroleuca's* vertical distribution on the shore is dictated by an inability to tolerate greater periods of low tide stress or through *L. digitata's* competitive dominance at higher tidal heights. Indeed, *L. ochroleuca* is found in much more varied habitats at lower latitudes that are currently occupied by *L. digitata* in Britain (e.g. tide pools & exposed coasts) (Pereira, Engelen, Pearson, Valero, & Serrão, 2015). The acquisition of such knowledge will allow for more accurate predictions of the structure and functioning of these communities in a future warmer world.

Optimal growth and maximal survival temperatures of macroalgae are well studied (Bolton & Anderson, 1987; Bolton & Lüning, 1982; Hargrave et al., 2016; Lüning, 1984; Lüning & Freshwater, 1988; Orfanidis, 1991; Simonson, Metaxas, & Scheibling, 2015; Tom Dieck, 1992) but the effects of consecutive low tide exposures remain relatively unknown (but see Pereira et al., 2015). This is surprising as low tide stress has long been known to be important in determining the vertical and latitudinal distributions of intertidal organisms (Dring, 1982; Evans, 1948) and increases in aerial temperatures can cause shifts in macroalgae distributions (Harley & Paine, 2009; Harley et al., 2012; Martínez et al., 2012; Ugarte, Critchley, Serdynska, & Deveau, 2009). Here we examined the potential for *L. ochroleuca* to move into niches left behind by *L. digitata* as its biogeographic range contracts in response to warming. Firstly, we determined the thermal tolerance of both species by subjecting individuals to a single temperature shock using a traditional metric of stress, upregulation of *Hsp70* across a range of temperatures. Secondly, we used chlorophyll florescence (*F*_v_ */F*_m_ *)* to investigate the resistance and resilience of each species to consecutive low tide scenarios. Understanding tolerances to consecutive low tide stress of these two species will provide important insight into how climate change can alter range edge dynamics whilst also providing foresight into the future species composition of kelp‐dominated communities on Northeast Atlantic rocky shores.

## 2 MATERIALS AND METHODS

Mature *L. digitata* and *L. ochroleuca* sporophytes (*n* = 5) were collected on a low spring tide at St Mawes Bay, Cornwall, UK (50°9′24.4764″ N, 5°1′4.1628″ W) in April and September 2015. Heat shock experiments (qPCR) were conducted in April and consecutive stress assays were conducted in September. All individuals were taken from the overlapping zone where both species directly coexist (*c*. 0.4 m above chart datum). Individuals were transported to the laboratory in cool dark containers and held in aerated recirculating tanks for *c*. 7 days under photosynthetic flux density of *c*. 20 μmol m^−2^ s^−1^ (12:12 hr). Holding tanks were maintained at ambient sea temperatures, 9°C (April) and 15°C (September). Following well‐established methods in seaweed photophysiology and gene expression studies (García‐Mendoza & Colombo‐Pallotta, 2007; Henkel & Hofmann, 2008; Jueterbock et al., 2014; Pearson, Lago‐Leston, & Mota, 2009; Rothäusler, Gómez, Karsten, Tala, & Thiel, 2011), we excised discs of tissue (diameter: 27 mm, area: 11.45 mm^2^) from each kelp, using a cork borer with discs left for 1 hr before being used in stress assays. To establish that excised discs responded similar to whole kelps, we measured *F*_v_ */F*_m_ values (a measure of stress see below), which were similar across tissue types.

### 2.1 Heat shock response

When environmental stresses cause proteins to denature and aggregate, organisms upregulate a suite of chaperone proteins known as Heat Shock Proteins (Hsps). These Hsps preserve normal cell function by ensuring appropriate protein folding during translation (Frydman, 2001), membrane stability, and transport (Hartl & Hayer‐Hartl, 2002) and protein refolding (Hendrick & Hartl, 1993). The level of Hsp transcript is a proxy for cellular damage and quantifying it across a range of experimental treatments allows powerful inferences regarding stress tolerance to be made. The heat shock response (HSR) is the induction of Hsps in response to temperature shocks (Parsell & Lindquist, 1993). Induction follows a bell shaped curve and specific thermal set points along this curve (*T*_peak_ and *T*_off_) indicate different important aspects of thermotolerance (Tomanek & Somero, 1999, 2002). *T*_peak_ represents an organism's maximum thermal tolerance as past this point protein synthesis can no longer keep up with the increasing thermal insult. *T*_off_ is an ultimate upper limit in the functioning of the translational machinery (Barua & Heckathorn, 2004; Tomanek, 2010). Both *T*_peak_ and *T*_off_ are fixed set points of the HSR and are not influenced by previous thermal history (Barua & Heckathorn, 2004). Therefore, seasonal differences in sampling periods between HSR experiments (spring) and chlorophyll florescence (summer) will not affect methodological comparisons.

To compare the HSR of *L. digitata* and *L. ochroleuca n* = 5 kelp discs per treatment were heat shocked at one of seven temperatures (8°C, 12°C, 16°C, 20°C, 24°C, 28°C or 32°C) for 1 hr. Discs were held in individual 100 ml sample pots in thermostatically controlled 35 L water baths with recirculating, aerated seawater. Discs were then removed, blotted dry and snap frozen in liquid N_2_ and stored at −80°C until RNA extraction.

### 2.2 RNA extraction and qPCR

Total RNA was extracted following a protocol from (Pearson, Lago‐Leston, Valente, & Serrão, 2006). All glassware and consumables were treated with RNAseAway (Ambion, UK) and rinsed with DEPC‐treated water before use. All reagents were molecular grade and RNAase free. Half a disc (*c*. 300 mg) of heat shocked kelp tissue was ground to a fine powder under liquid N_2,_ using a pestle and mortar, placed in an extraction buffer (100 mM Tris, 50 mM EDTA, 2 M NaCl and 2% CTAB pH 7.5), vortexed and left at room temperature (RT) for 10–15 min. To minimise endogenous RNase activity, DTT was added (from 1 M stock) to the extraction buffer immediately prior to use to a final concentration of 50 mM. Total RNA was extracted from the resultant homogenate by chloroform extraction. One ml of chloroform:Isoamyl alcohol (24:1 v/v) was added, vortexed vigorously, centrifuged at 12,000 g for 20 min at 20°C and the upper aqueous phase transferred to a new tube. Absolute EtOH was added (0.3 volumes) and gently mixed by rocking. Another chloroform extraction was then performed on this upper aqueous phase/EtOH mixture under the same conditions as the first. RNA was precipitated with 0.25 vol 12 M LiCl in the presence of 1% mercaptoethanol at −80°C for 30 min. Samples were centrifuged at 14,000 g for 30 min at 4°C and the pellet washed with 70% EtoH and recollected by centrifugation at 14,000 g for 10 min at RT. The RNA pellet was air‐dried and then suspended in 50 μl DEPC‐treated water. Contaminating gDNA was digested, using 2U DNAse 1 (Turbo‐DNAse free) (Ambion, UK) following the manufacturer's instructions and stored at −80°C. Total RNA (100 ng) was reversed transcribed using High Capacity cDNA Reverse Transcription Kit (Applied Biosystems) according to manufacturer's instructions and using random hexamer primers.

In order to determine the sequence of the *Hsp70* gene for *L. ochroleuca* and *L. digitata,* PCR primers were designed based on *Saccharina japonica* (accession no FJ375359.1). PCRs were run in 20 μl volumes, using Bioline MyTaq Red PCR mix with 100 μM F and R primers and 1 μl cDNA template. Cycling parameters were 5 min at 95°C, followed by 40 cycles of 15 s at 95°C, 60 s at 55°C and 60 s at 72°C and a final extension of 10 min at 72°C. Amplicons were resolved on 1.5% agarose gels and products of the expected size were excised and extracted, using Bioline Isolate II clean‐up columns. All products were subjected to direct, Sanger sequencing in‐house.

primer 3 software (Life Technologies) was used to design all qPCR primers. *Hsp70* primers were based on the consensus sequences for *L. ochroleuca* and *L. digitata*. For *L. digitata* gene expression normalisation, two reference genes were used, 18s ribosomal RNA (accession no. JX442505) and Rubisco large subunit (accession no. AY157696). For *L. ochroleuca* expression normalisation only used 18s Ribosomal RNA (Table 1).

**Table 1:** qPCR primer sequences used for assays on heat shocked *Laminaria digitata* and *Laminaria ochroleuca*

	Forward	Reverse
*L. digitata*		
*Hsp70*	GCTGCGAGTCGTTGAAGTA	TGGTGCTCGTGAAGATGAAG
*18s rRNA*	CGGAAGGATCATTACCGAAA	CCCAACTTCGCATAACGAAT
*Rubisco*	GACATGGATTGGGCATCTCTT	GTAGAACCACATCGTCACCTA
*L. ochroleuca*		
*Hsp70*	GAACTTGCGCTTGAACTCTTG	GACGATCGAGGAGGGTATCT
*18s rRNA*	GATGAAGAACGAGCGAAATG	GTCAACAGACACTCCGACAA

Quantitative PCR reactions were run in triplicate on 384 well plates on an Applied Biosystem Quant Studio 120‐Flex platform, with each plate containing all targets and housekeeping genes for each species. Each well contained 1 μl of cDNA in 10 μl SYBR green reactions (Bioline Sensifast Lo‐Rox kit). The PCR amplification protocol consisted of 95°C for 2 min followed by 40 cycles of 95°C for 5 s and 60°C for 15 s (for all assays). Amplification specificity was verified by melt curve analysis (from 60°C to 95°C) and agarose gel electrophoresis. Four 10‐fold serial dilutions of cDNA product were used to verify that all amplification reactions had efficiencies in the range of 90%–105%. No reverse transcriptase and no template controls were performed for each assay plate to ensure no contamination from gDNA was present. Relative mRNA levels were calculated as follows: Firstly, the difference in Ct values from *Hsp70* and the internal reference gene were calculated (ΔCT). The ΔCT value was then subtracted by the ΔCT of the control (8°C) from each individual (ΔΔCT). Relative expression of *Hsp70* was then calculated by e^(−ΔΔCT)^.

### 2.3 Consecutive stress experiments

The experimental design for consecutive stress assays was based on field observations. Both species are found on the low shore and are exposed to low tide stress together only on spring tides. Aerial exposure usually last *c*. 1 hr over each low tide period for four days before becoming immersed until the next spring tidal cycle.

Consecutive stress assays (*n* = 5) were conducted on two discs of tissue a mean value of which was used in all further analysis. Individual discs were placed under one of a number of different conditions for 1 hr, to simulate the low tide and then placed back into the control tanks (15°C) to simulate the returning tide. For immersed treatments, discs were held in individual 100 ml sample pots in thermostatically controlled 35 L water baths with recirculating, aerated seawater. After 24 hrs recovery in control tanks, discs were again exposed to their respective 1 hr treatments. In total, four simulated tidal cycles were performed on the same disc. Recovery was subsequently monitored daily for three days. Stress measurements (*F*_v_/*F*_m_ see below) were taken directly before and after each “low tide” treatment.

Treatments consisted of either emersed or immersed conditions and tested tolerance to either high or low temperatures. Conditions were: control at 15°C, immersion at 24°C, 28°C, 32°C (simulating summer tidal pools), 0°C and 4°C (simulating winter tidal pools), emersion at 24°C, 28°C, and 32°C (simulating summer low tide aerial exposure) and −5°C (simulating severe winter ground frost). These temperature ranges were chosen to represent realistic field exposures. Low shore tidal pools in the study area can reach 28°C in summer (Martins, Hawkins, Thompson, & Jenkins, 2007) while blades of *L. ochroleuca* can be subjected to temperatures of >30°C in tidal pools in its southern range (Engelen et al., 2008). Similarly aerial temperatures can already reach 32°C in summer and ground frosts are encountered during winter spring tides (Moore, unpub).

The experimental design allows clear interspecific comparisons to be made for different types of immersion and emersion stress, yet some caution should be taken when generalising to field conditions. For example, only temperature was elevated in warm‐emersed treatments whereas in natural settings a number of environmental factors contribute to low tide stress including relative humidity, wind speed and solar radiation. Moreover, the abrupt temperature shocks used in this experiment are realistic for emersion stresses but are more rapid than the gradual warming seen in tidal pools. However, such shocks still allow direct comparisons of interspecific thermal tolerance (Henkel & Hofmann, 2008; Jueterbock et al., 2014; Smolina, Kollias, Jueterbock, Coyer, & Hoarau, 2016).

Stress was measured, using pulse amplitude modulated (PAM) fluorometry (Walz Diving PAM, Germany). The maximum quantum yield of PSII (ratio of photochemical quenching (*F*_v_) to total fluorescence from closed PSII reaction centres (*F*_m_)) is proportional to its efficiency (Butler, 1978) and *F*_v_/*F*_m_ is an established method to quantify temperature stress in seaweeds (Jueterbock et al., 2014; Pearson et al., 2009; Saada et al., 2016). All *F*_v_/*F*_m_ values were dark adapted for 15 min. The results for each species were normalised to one using the initial *F*_v_/*F*_m_ values taken prior to the start of any treatments. This allowed small intrinsic interspecific differences in starting *F*_v_/*F*_m_ to be accounted for.

For high temperature emersion treatments the level of desiccation experienced at each time point was expressed as relative water content (RWC %). Initial wet weights of fully hydrated discs were obtained at the start of the experiment prior to any experimental treatments (FW) and subtracted from their dry weights (DW) (estimated based on wet: dry weight of discs of both kelps, *n* = 12 for both *L. digitata* and *L. ochroleuca*, taken independently and dried at 60°C for 48 hrs). Wet weight of kelp discs at each experimental time point (IW) (pre‐ and post‐treatments) was subtracted from the dry weight and then divided by FW‐DW. The resulting value was multiplied by 100.

RWC=[(IW−DW)/(FW−DW)]×100

IW is wet weight of treated kelp; FW is initial wet weight of fully hydrated kelp (pre‐treatments); DW is dry weight of kelp disc.

### 2.4 Statistical analysis

Differences in upregulation of *Hsp70* to common garden temperature stress between species was measured, using univariate permutational ANOVA and the PERMANOVA module (Anderson, 2001) within primer 6 software (Clarke & Gorley, 2006). The model included the two fixed factors; species (2 levels: *L. digitata*,* L. ochroleuca*) and temperature (7 levels: 8°C, 12°C, 16°C, 20°C, 24°C, 28°C & 32°C). Permutations (9,999 under a reduced model) were conducted on a similarity matrix constructed from Euclidean distances between untransformed data (relative *Hsp70* expressions). When conducting PERMANOVA analysis on univariate data using Euclidean distances, outputs (pseudo‐*F*‐statistics) are analogous to traditional least‐squares ANOVA, without the same severity of assumptions regarding data distributions and homogeneity of variance (Anderson, 2001; McArdle & Anderson, 2001).

*F*_v_/*F*_m_ values for consecutive stress assays were analysed using repeated measures analysis of variance (RM ANOVA) in SPSS v.22 (IBM). The models had three factors: species (2 levels: *L. digitata, L. ochroleuca*), temperature (heat emersion and immersion—4 levels: 15°C, 24°C, 28°C & 32°C; cold emersion—2 levels: 15°C & −5°C and; cold immersion—3 levels: 15°C, 4°C & 0°C) and time (11 time points). Relative water content values for heat emersion temperatures were also measured using RM ANOVA with three factors: species (2 levels: *L. digitata* & *L. ochroleuca*), temperature (4 levels: 15°C, 24°C, 28°C, 32°C) and time (9 time points). Where the assumption of sphericity was violated (Mauchly's test) degrees of freedom were corrected, using Greenhouse–Geisser estimates of Sphericity. Also, where significant differences were found (at *p* < .05) post hoc analysis was employed to facilitate interpretation of the RM ANOVA results.

## 3 RESULTS

### 3.1 Heat shock response

**Figure 1:**
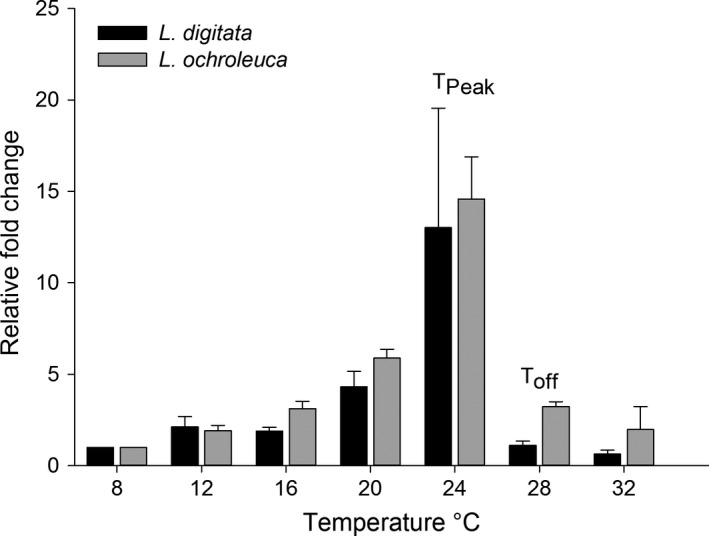
Mean (+1 *SE*) relative upregulation of *Hsp70* of *Laminaria digitata* and *Laminaria ochroleuca* from St Mawes Bay, UK to 1 hr heat shock treatments (*n* = 5). *Laminaria digitata* normalised to 2 reference genes (ribosomal RNA and Rubiscos large subunit), *L. ochroleuca* normalised to 1 reference gene (Rubisco large subunit)

Upregulation of *Hsp70* to the temperature treatments followed a clear bell shaped response for both species (Figure 1). There was no significant interaction between species and temperature (PERMANOVA *F*_(6,56)_ = 0.10 *p* = .99) (Table S1). In both species, thermal set points of the HSR were the same, *T*_peak_ 24°C and *T*_off_ 28°C (Figure 1).

### 3.2 Consecutive heat shock

#### 3.2.1 Immersed (heat shock)

**Figure 2:**
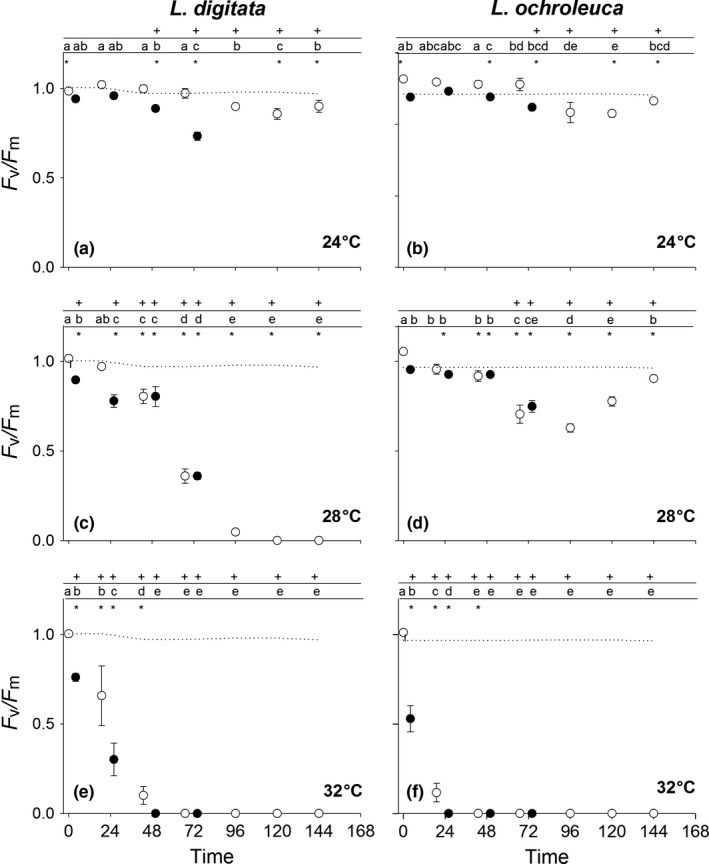
Mean (±1 *SE*) *F*_v_/*F*_m_ values (normalised to 1) for *Laminaria digitata* (left) and *Laminaria ochroleuca* (right) from St Mawes Bay, UK in response to consecutive 1 hr immersion exposures at (a + b) 24°C, (c + d) 28°C and (e + f) 32°C (*n* = 5). Closed symbols are taken immediately after treatments. Initial open symbol represents *F*_v_/*F*_m_ prior to any experimental treatment. Subsequent open symbols are taken 24 hrs after treatments to track daily recovery. Dotted line represents mean *F*_v_/*F*_m_ for 15°C controls. + represents significant difference from control for that temperature treatment. * represents significant difference between species for that temperature treatment. Different letters represent significant differences from means within each species and treatment

Normalised *F*_v_/*F*_m_ values varied between time, species and temperature (RM ANOVA: Time × Species × Temperature *F*_(10.3,109.6)_ = 16.2, *p* < .0001) (Table S2). A general pattern of a small reduction in *F*_v_/*F*_m_ with each 24°C heat shock followed by recovery was observed for both species (Figure 2a,b). In initial heat shock treatments, these were not significantly different from the control. However, by the 3rd heat shock for *L. digitata* and 4th for *L. ochroleuca* values were significantly different to control discs held at 15°C. Whilst not returning to control values after 3 days of recovery both species showed patterns of resilience, indicating that heat stress was reversible.

At 28°C, species showed clear differences in their responses from the first heat shock treatment. Initial treatments (shocks 1–3) only caused minor reductions in *F*_v_/*F*_m_ values for *L. ochroleuca* followed by a considerable reduction at shock 4 (Figure 2d). However, there were signs of recovery after each 24 hr period and *F*_v_/*F*_m_ levels almost reached those of controls after 3 days recovery. On the other hand, *L. digitata* was only able to recover from its first shock at 28°C. By the fourth cycle values had reached <40% of the control and all discs non‐viable 24 hrs after the final shock (Figure 2c).

Neither species showed any ability to tolerate heat shocks of 32°C with all discs non‐viable by the second cycle (Figure 2e,f).

#### 3.2.2 Immersed (cold shock)

**Figure 3:**
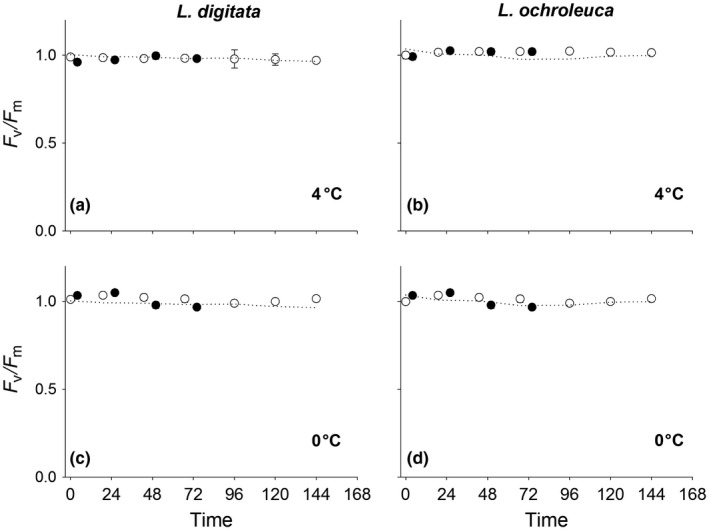
Mean (±1 *SE*) *F*_v_/*F*_m_ values (normalised to 1) for *Laminaria digitata* (left) and *Laminaria ochroleuca* (right) from St Mawes Bay, UK in response to consecutive 1 hr immersion shocks at (a + b) 4°C and (c + d) 0°C (*n* = 5). Closed symbols are taken immediately after treatments. Initial open symbol represents *F*_v_/*F*_m_ prior to any experimental treatment. Subsequent open symbols are taken 24 hrs after treatments to track daily recovery. Dotted line represents 15°C controls

*F*_v_/*F*_m_ values did not vary significantly between species, temperature and time (RM ANOVA: Time × Species × Temperature *F*_(6.3,75)_ = 1.9 *p* = .07) (Table S4). At 4°C, there was no pattern of reduced *F*_v_/*F*_m_ values pre‐ or post‐treatment (Figure 3a,b). At 0°C, treatments began to affect *F*_v_/*F*_m_ values but differences were not significantly different from control values, suggesting that these conditions were non‐stressful (Figure 3c,d).

#### 3.2.3 Emersed (heat shock)

**Figure 4:**
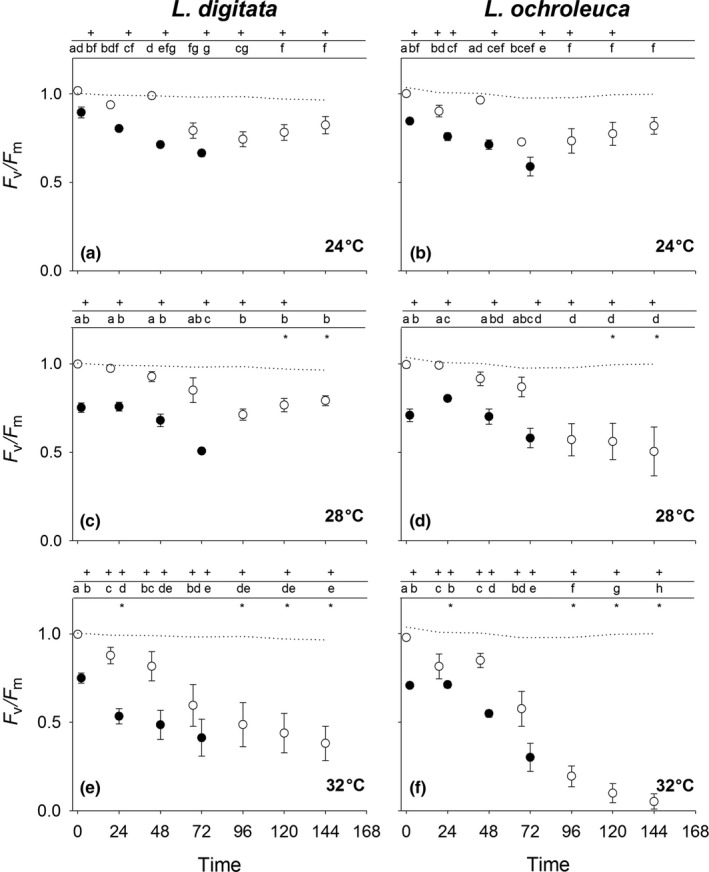
Mean (±1 *SE*) *F*_v_/*F*_m_ values (normalised to 1) for *Laminaria digitata* (left) and *Laminaria ochroleuca* (right) from St Mawes Bay, UK in response to consecutive 1 hr emersion shocks at (a + b) 24°C, (c + d) 28 and (e + f) 32°C (*n* = 5). Closed symbols are taken immediately after treatments. Initial open symbol represents *F*_v_/*F*_m_ prior to any experimental treatment. Subsequent open symbols are taken 24 hrs after treatments to track daily recovery. Dotted line represents 15°C controls. + represents significant difference from control. *represents significant difference between species. Different letters represent significant differences from means within each species and treatment

*F*_v_/*F*_m_ values varied significantly between species, temperature and time (RM ANOVA: Time × Species × Temperature *F*_(8.7,92.3)_ = 2.27 *p* = .03) (Table S3). At 24°C both species showed similar patterns of response with an almost full recovery from exposures 1 and 2. However, after the third exposure recovery was impeded. A pattern of slow recovery was seen in both species yet neither reached close to control values after 72 hrs (Figure 4a,b).

At 28°C a similar pattern of depression of *F*_v_/*F*_m_ values followed by recovery was seen in both species for the first three cycles. However, recovery from the fourth cycle was only evident for *L. digitata* (Figure 4c) with values for *L. ochroleuca* still declining after 72 hrs (Figure 4d).

At 32°C a similar pattern to those seen above was observed for the first two cycles, with recovery occurring after 24 hrs. However, after the third cycle recovery was considerably reduced. During the recovery period *F*_v_/*F*_m_ values continued to decline in both species (Figure 4e,f). This was most marked in *L. ochroleuca*.

### 3.3 Water loss

**Figure 5:**
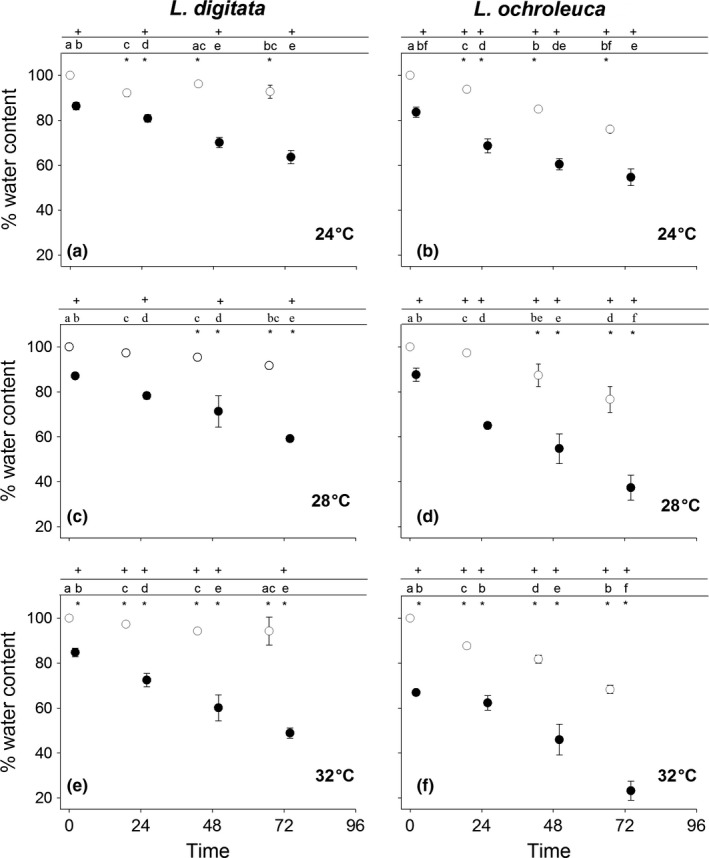
Mean (±1 *SE*) relative water content of *Laminaria digitata* (left) and *Laminaria ochroleuca* (right) from St Mawes Bay, UK in response to four consecutive 1 hr emersion exposures at (a + b) 24°C, (c + d) 28°C and (e + f) 32°C (*n* = 5). Initial open symbol represents *F*_v_/*F*_m_ prior to any experimental treatment. Closed symbols are taken immediately after treatments. Initial open symbol represents *F*_v_/*F*_m_ prior to any experimental treatment. Subsequent open symbols are taken 24 hrs after treatments to track daily recovery. + represents significant difference from control. * represents significant difference between species. Different letters represent significant differences from means within each species and treatment

Water loss varied significantly between time, species and temperature (RM ANOVA: Time × Species × Temperature *F*_(9.6,102)_ = 2.63 *p* = .007) (Table S6). Kelp subjected to aerial temperatures followed a clear cycle of reduced relative water content (RWC) after each exposure, followed by an increase after 24 hrs (Figure 5). The magnitude of reduction in RWC increased with increasing temperatures while the ability to recover to pre heat shocked water content decreased. A cumulative reduction in RWC with each consecutive treatment was observed across all temperatures for both species. The rate of water loss was consistently greater in *L. ochroleuca,* which also failed to rehydrate fully after 24 hr recovery. In comparison, *L. digitata* was generally almost fully hydrated after the recovery period (Figure 5).

#### 3.3.1 Emersed (cold shock)

**Figure 6:**
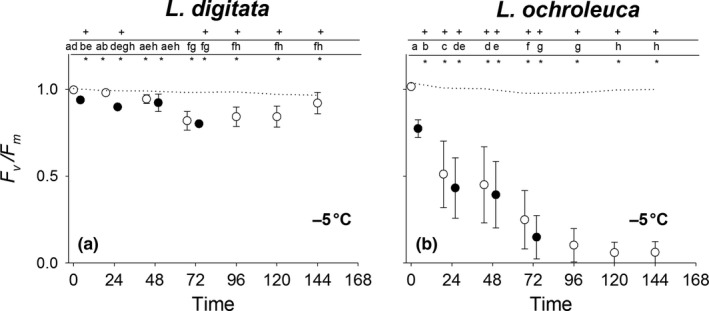
Mean (±1 *SE*) *F*_v_/*F*_m_ values (normalised to 1) for *Laminaria digitata* (left) and *Laminaria ochroleuca* (right) from St Mawes Bay, UK in response to consecutive 1 hr emersion shocks at −5°C. Closed symbols are taken immediately after treatments. Initial open symbol represents *F*_v_/*F*_m_ prior to any experimental treatment. Subsequent open symbols are taken 24 hrs after treatments to track daily recovery. Dotted line represents 15°C controls. + represents significant difference from control. * represents significant difference between species. Different letters represent significant differences from means within each species and treatment

*Laminaria digitata* and *L. ochroleuca* exhibited very different tolerances to −5°C emersion stress (RM ANOVA: Time × Species × Temperature *F*_(1.7,27.1)_ = 8.436 *p* = .002) (Table S5). *Laminaria digitata* showed reduced *F*_v_/*F*_m_ values with each exposure recovering to near control levels after 24 hrs recovery for the first two cycles. After the third exposure, values were still depressed after 24 hrs and a subsequent exposure resulted in no further reduction. Following 72 hrs, recovery values had returned to almost that of control values indicating that this freezing stress was reversible (Figure 6a). *Laminaria ochroleuca* was much less tolerant to this treatment with large reductions in *F*_v_/*F*_m_ values from the initial exposure which dropped further after 24 hrs recovery. After the four exposures, values were down to 15% (±12.3% *SE*) of the control and there were no signs of recovery after 72 hrs (Figure 6b).

## 4 DISCUSSION

### 4.1 Interspecific difference in consecutive low tide tolerance

The two species exhibited varied responses to consecutive immersed heat shocks with the warmer water species, *L. ochroleuca,* better equipped to deal with higher temperature treatments. At 24°C, a progressive erosion of resilience was seen in *L. digitata* that was not observed in *L. ochroleuca*. By the final treatment at 28°C, all *L. digitata* were non‐viable whereas *L. ochroleuca* were able to return to control values after 3 days of recovery. Interestingly, a similar lethal response at 28°C was not observed in *L. digitata* during emersed treatments which is likely a product of evaporative cooling reducing the tissue temperature or desiccation inducing an ametabolic state. Both species differed in their abilities to tolerate stress related to emersion. Most strikingly, *L. ochroleuca* showed no tolerance to even a single freezing exposure. Indeed, anecdotal evidence supports this with reports that over mild winters, *L. ochroleuca* progressively advances up the shore becoming more abundant at higher shore heights, until a severe ground frost results in rapid die off of in the intertidal (G. Boalch, pers. comm.). *Laminaria ochroleuca* was also less equipped to tolerate elevated aerial temperatures, which seemed related to the greater water loss experienced resulting in more severe desiccation. *Laminaria digitata* has been shown to recover from 60% water loss (Dring, 1982), which was also evident in this study. Whilst desiccation tolerance levels are unavailable for *L. ochroleuca*, it was where water loss fell below *c*. 60% that recovery was not possible.

Climate change in the UK is predicted to result in less summer cloud, increased air temperatures and sunny days, while winter ground frosts will become more infrequent (Jenkins et al., 2009). This may result in *L. ochroleuca's* maximum tidal height switching from being cold limited in the winter to desiccation limited in the summer. Therefore, the habitat vacated by *L. digitata* may remain unoccupied by *L. ochroleuca* as conditions will still remain outside of its fundamental niche. However, it should be noted that conditions may change asymmetrically. Night temperatures are rising faster than day temperatures (Davy, Esau, Chernokulsky, Outten, & Zilitinkevich, 2017) so advancement up the shore may be possible at least for a period of time. One habitat where *L. ochroleuca* may be able to expand into is tidal pools. Tidal pools in SW England can already reach >28°C in summer (Martins et al., 2007), a temperature that results in considerable stress to *L. digitata* but not *L. ochroleuca*. Currently, only *L. digitata* occupies these habitats suggesting *L. ochroleuca* is being competitively excluded. As temperatures rise and *L. digitata* becomes less competitively dominant or vacates these tidal pools, *L. ochroleuca* will face few barriers to expanding its realised niche to encompass these habitats.

### 4.2 Comparing the heat shock response and *F*_v_/*F*_m_ (single exposures)

We observed a clear disparity between the HSR and *F*_v_ */F*_m_ as metrics for stress. No interspecific differences in thermal set points of the HSR were found but differences were observed in *F*_v_ */F*_m_ to similar single stress treatments. For example, *L. digitata* were able to recover from exposures that represent *T*_peak_ and *T*_off_ of the HSR, while *L. ochroleuca* showed no deviation away from control values. This is surprising given that *T*_peak_ and *T*_off_ are thought to represent the very upper limits of a species tolerance (Barua & Heckathorn, 2004).

Mismatches between chlorophyll florescence and the HSR have been observed before (Jueterbock et al., 2014) and may be due to the photosynthetic apparatus having other protective mechanisms in place, allowing for greater thermotolerance compared to the rest of the cell (Downs, Mueller, Phillips, Fauth, & Woodley, 2000). For example, the small HSP, cp‐sHSP, is known to play an important role in protecting photosystem II during heat stress, and production levels are related to thermotolerance in higher plants (Neta‐Sharir, Isaacson, Lurie, & Weiss, 2005; Shakeel, Haq, Heckathorn, & Luthe, 2012). Chlorophyll florescence may also differ from Hsp quantification as it measures different aspects of heat stress and not just symptoms of protein damage. For example, stress induced uncoupling of enzymes and metabolic pathways can cause the accumulation of reactive oxygen species that can lead to oxidative stress affecting *F*_v_/*F*_m_ values (Liu & Pang, 2010). Plants and seaweeds can upregulate genes with anti‐oxidative functions during periods of heat stress to protect the photosystems (Collén, Guisle‐Marsollier, Léger, & Boyen, 2007) and as such maintain photosynthetic function. Thus, different pressures and mechanisms may affect the two metrics differently and they should not be used interchangeably. Moreover, measuring the HSR alone is not sufficient to fully determine the levels of stress affecting macrophytes as they are likely to be more tolerant than levels of *T*_peak_, and even *T*_off_ would suggest.

### 4.3 Single vs. consecutive exposures

Intertidal stress studies have overwhelmingly focussed on absolute limits from single treatments with cumulative effects largely being ignored. We identified clear interspecific differences in tolerances to low tide scenarios that were only apparent after consecutive exposures. For example, if a single treatment had been conducted on *L. digitata* at 28°C, we would conclude that such temperature shocks would be non‐lethal as full recovery was attained within 24 hrs. Moreover, when comparing this to *L. ochroleuca* we may conclude they exhibit similar tolerances. However, after the final treatment, all *L. digitata* were non‐viable whereas *L. ochroleuca* had returned to near control values. Combined with data from the HSR indicating similar tolerances, measuring a single treatment would lead to potentially misleading interpretations.

Environmental Niche Models (ENMs also called distribution, envelope and bioclimatic models) are the most widely used tools to predict the impact of climate change in species distributions. The majority of ENMs determine where a species can exist in the future based on the conditions they exist in now. However, in recent years there has been an attempt to base ENMs on mechanistic cause and effect relationships with environmental variables (Jordà, Marbà, & Duarte, 2012; Martínez, Arenas, Trilla, Viejo, & Carreño, 2015; Sunday et al., 2012). Modellers seek to link critical temperature thresholds (e.g. CT_max_ and CT_min_) with current and future warming scenarios. Here we show an erosion of resilience leading to mortality at temperatures lower than CT_max_ with consecutive exposures. Therefore, such models may underestimate the effect of future warming on species distributions, with local extinctions potentially occurring at lower temperatures. This is especially true for intertidal and shallow subtidal species that experience discrete consecutive exposures on a daily basis or during a set of exposures that coincide with low spring tides. In such cases, smaller increases in temperatures or discrete “heatwave” events (Hobday et al., 2016), could result in consecutive exposures lower than CT_max_ but great enough to result in considerable stress and subsequent mortality. Therefore, the effect of consecutive exposures on thermal tolerance should be factored into predictive models wherever possible.

## 5 CONCLUSION

Our study provides insight into whether a range expander can replace a range contractor at the interface where they coexist within a biogeographic boundary zone. We show that cumulative stress is a key factor in determining interspecific differences in stress physiology. Such knowledge serves to increase our understanding of the processes driving species redistributions, which is comparatively lacking for marine organisms. However, some caution should be taken when generalising results to the wider region. Although the magnitude of difference between species is quite striking and unlikely to arise from site specific local adaptations such factors cannot be discounted, as only a single site and time point were sampled. Moreover, we have only investigated physiological differences between species and species interactions (e.g. facilitation) have not been accounted for. Such interactions may be important in further elucidating species responses to warming.

## AUTHORS’ CONTRIBUTIONS

N.G.K., P.J.M. and D.C.W. conceived the ideas and designed methodology; N.G.K. and L.S.H. collected the data; N.G.K. and P.J.M. analysed the data; all authors contributed to data interpretation; N.G.K. led the writing of the manuscript, with equal input from all authors. All authors contributed critically to the drafts and gave final approval for publication.

## DATA ACCESSIBILITY

Data available from Figshare online repository https://figshare.com/s/c2ad62334bdec04eb976 (King et al., 2017).
