# Supplementary material for: Cumulative stress restricts niche filling potential of habitat‐forming kelps in a future climate
Source: Funct Ecol. 2017 Sep 25;32(2):288–99. doi: 10.1111/1365-2435.12977 (PMC5856065; doi:10.1111/1365-2435.12977)
Supplement: Supplementary file 1 [file FEC-32-288-s001.pdf]

Functional Ecology

Changing Kelp Distributions in a Warmer World

Nathan G. King, David C. Wilcockson, Richard Webster, Dan A. Smale, Laura S. Hoelters, Pippa J. Moore

Where species can exist is largely determined by the temperature range they can tolerate. This is known as their thermal niche and climate change is changing where these niches are found in nature. At equatorward edges of species distributions conditions may now, or soon, be too hot and populations will die off, while at the poleward edge new habitat may become available for colonisation as cold limits are lifted.

Coastal species such as kelps are particularly sensitive to environmental change and the ecological effects of changing distributions can impact the whole ecosystem, as other species depend on them for food and shelter. In the English Channel a dynamic scenario exists where two kelp species are undergoing opposing range migrations. Oarweed (*Laminaria digitata*), a cooler-water kelp, is contracting while the warmer-water golden kelp (*Laminaria ochroleuca*), is expanding. *L. digitata* currently inhabits a zone immediately above *L. ochroleuca* which is inherently more stressful owing to greater time spent out of the water at low tide. In order to determine the capacity of *L. ochroleuca* to move into the niche currently occupied by *L. digitata* as it contracts, we subjected both species to a range of single and consecutive treatments, replicating low tide stress extremes experienced during summer and winter. Our results suggest that *L. ochroleuca* will not be able to advance up

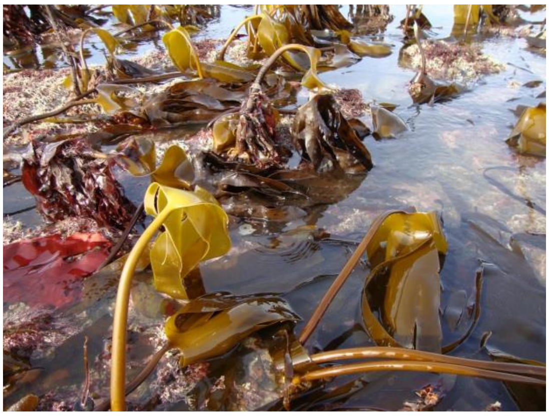

Photograph provided by authors.

the shore as desiccation stress in summer will be too great, but it will be able to move into tidal pools. This means that for the most part, *L. digitata*'s current niche will go unoccupied by a similar kelp species. The impacts of this could be far reaching and need to be fully explored.

Importantly, many of the differences between these two species were only observed after consecutive treatments which are more reflective of the conditions faced in nature over many tidal cycles. Most intertidal stress studies have overlooked this potentially fundamental issue. As such, we recommend that future studies incorporate cumulative stress into their experimental design.
