# Supplementary material for: Cumulative stress restricts niche filling potential of habitat‐forming kelps in a future climate
Source: Funct Ecol. 2017 Sep 25;32(2):288–99. doi: 10.1111/1365-2435.12977 (PMC5856065; doi:10.1111/1365-2435.12977)
Supplement: Supplementary file 2 [file FEC-32-288-s002.docx]

**Supplementary Information:**

**Table S1.** Results of a univariate Permutational ANOVA to test for differences in hsp70 upregulation to common garden stress in the kelps *Laminaria digitata* and *Laminaria ochroleuca* (8–32 ^o^C). Permutations (4999) were conducted under a reduced model and were based on matrices derived from Euclidean distances, with Temperature and Species as fixed factors.

| Source | df | SS | MS | F | P | Den df |
| --- | --- | --- | --- | --- | --- | --- |
| Temperature | 6 | 1231 | 205 | 11.3 | 0.0001 | 56 |
| Species | 1 | 20.9 | 20.8 | 1.1 | 0.33 | 56 |
| Temperature X Species | 6 | 11.3 | 1.9 | 0.1 | 0.99 | 56 |

**Table S2.** Results of a repeated measures ANOVA to test for differences in normailsed *F_v_/F_m_* values in response to consecutive 1 hour immersion heat shocks (15, 24, 28 & 32 ^o^C). The model has three factors: Species, Temperature and Time. Significant values (p < 0.05) are highlighted in bold.

| Source | df | SS | MS | F | p |
| --- | --- | --- | --- | --- | --- |
| Time | 3.4 | 9.3 | 2.7 | 231.6 | **<0.0001** |
| Time x Species | 3.4 | 1.4 | 0.3 | 33.3 | **<0.0001** |
| Time x Temperature | 10.2 | 9.2 | 10.2 | 75.7 | **<0.0001** |
| Time x Species x Temperature | 10.2 | 2.0 | 10.2 | 16.24 | **<0.0001** |

**Table S3.** Results of a repeated measures ANOVA to test for differences in normailsed *F_v_/F_m_* values in response to consecutive 1 hour emersion heat shocks (15, 24, 28 & 32 ^o^C). The model has three factors: Species, Temperature and Time. Significant values (p < 0.05) are highlighted in bold.

| Source | df | SS | MS | F | p |
| --- | --- | --- | --- | --- | --- |
| Time | 2.8 | 6.5 | 2.3 | 67 | **<0.0001** |
| Time x Species | 2.8 | 0.43 | 0.15 | 4.4 | **0.006** |
| Time x Temperature | 8.6 | 3.82 | 0.44 | 13.1 | **<0.0001** |
| Time x Species x Temperature | 8.6 | 0.63 | 0.07 | 2.1 | **0.03** |

**Table S4.** Results of a repeated measures ANOVA to test for differences in normailsed *F_v_/F_m_* values in response to consecutive 1 hour cold immersion shocks (15, 0 & 4^o^C). The model has three factors: Species, Temperature and Time. Significant values (p < 0.05) are highlighted in bold.

| Source | df | SS | MS | F | P |
| --- | --- | --- | --- | --- | --- |
| Time | 3.1 | 0.04 | 0.01 | 5.7 | **0.001** |
| Time x Species | 3.1 | 0.02 | 0.01 | 3.0 | **0.036** |
| Time x Temperature | 6.3 | 0.07 | 6.3 | 5.4 | **< 0.0001** |
| Time x Species x Temperature | 6.3 | 0.03 | 6.3 | 2.0 | 0.074 |

**Table S5.** Results of a repeated measures ANOVA to test for differences in normailsed *F_v_/F_m_* values in response to consecutive 1 hour emersion shocks (15 & -5 ^o^C). The model has three factors: Species, Temperature and Time. Significant values (p < 0.05) are highlighted in bold.

| Source | df | SS | MS | F | P |
| --- | --- | --- | --- | --- | --- |
| Time | 1.7 | 1.8 | 1.1 | 16.4 | **< 0.0001** |
| Time x Species | 1.7 | 0.9 | 0.56 | 8.4 | **0.002** |
| Time x Temperature | 1.7 | 1.3 | 0.76 | 11.5 | **< 0.0001** |
| Time x Species x Temperature | 1.7 | 0.9 | 0.5 | 7.7 | **0.004** |

**Table S6.** Results of a repeated measures ANOVA to test for differences in relative water content (%) in response to consecutive 1 hour emersion shocks (24, 28 & 32 ^o^C). The model has three factors: Species, Temperature and Time. Significant values (p < 0.05) are highlighted in bold.

| Source | df | SS | MS | F | P |
| --- | --- | --- | --- | --- | --- |
| Time | 3.2 | 49506 | 15460 | 212 | **< 0.0001** |
| Time x Species | 3.2 | 1813 | 566 | 7.7 | **< 0.0001** |
| Time x Temperature | 9.6 | 18751 | 1952 | 26.8 | **< 0.0001** |
| Time x Temperature x Species | 9.6 | 1842 | 191 | 2.6 | **0.007** |
